# Comparing anterior gastropexy to no anterior gastropexy for paraesophageal hernia repair: a study protocol for a randomized control trial

**DOI:** 10.1186/s13063-022-06571-8

**Published:** 2022-07-30

**Authors:** K. E. Blake, S. J. Zolin, C. Tu, K. F. Baier, L. R. Beffa, D. Alaedeen, D. M. Krpata, A. S. Prabhu, M. J. Rosen, C. C. Petro

**Affiliations:** 1 Department of General Surgery, Digestive Disease and Surgery Institute, Cleveland Clinic Foundation, 9500 Euclid Avenue, A10-133, Cleveland, OH 44195 USA; 2 Department of Quantitative Health Sciences, Cleveland Clinic Foundation, 9500 Euclid Avenue, Cleveland, OH 44195 USA

**Keywords:** Paraesophageal hernia, Hiatal hernia, Recurrence, Gastropexy, Randomized control trial, Laparoscopic, Outcomes

## Abstract

**Background:**

More than half of patients undergoing paraesophageal hernia repair (PEHR) will have radiographic hernia recurrence at 5 years after surgery. Gastropexy is a relatively low-risk intervention that may decrease recurrence rates, but it has not been studied in a prospective manner. Our study aims to evaluate the effect of anterior gastropexy on recurrence rates after PEHR, compared to no anterior gastropexy.

**Methods:**

This is a two-armed, single-blinded, registry-based, randomized controlled trial comparing anterior gastropexy to no anterior gastropexy in PEHR. Adult patients (≥18 years) with a symptomatic paraesophageal hernia measuring at least 5 cm in height on computed tomography, upper gastrointestinal series, or endoscopy undergoing elective minimally invasive repair are eligible for recruitment. Patients will be blinded to their arm of the trial. All patients will undergo laparoscopic or robotic PEHR, where some operative techniques (crural closure techniques and fundoplication use or avoidance) are left to the discretion of the operating surgeon. During the operation, after closure of the diaphragmatic crura, participants are randomized to receive either no anterior gastropexy (control arm) or anterior gastropexy (treatment arm). Two hundred forty participants will be recruited and followed for 1 year after surgery. The primary outcome is radiographic PEH recurrence at 1 year. Secondary outcomes are symptoms of gastroesophageal reflux disease, dysphagia, odynophagia, gas bloat, regurgitation, chest pain, abdominal pain, nausea, vomiting, postprandial pain, cardiovascular, and pulmonary symptoms as well as patient satisfaction in the immediate postoperative period and at 1-year follow-up. Outcome assessors will be blinded to the patients’ intervention.

**Discussion:**

This randomized controlled trial will examine the effect of anterior gastropexy on radiographic PEH recurrence and patient-reported outcomes. Anterior gastropexy has a theoretical benefit of decreasing PEH recurrence; however, this has not been proven beyond a suggestion of effectiveness in retrospective series. If anterior gastropexy reduces recurrence rates, it would likely become a routine component of surgical PEH management. If it does not reduce PEH recurrence, it will likely be abandoned.

**Trial registration:**

ClinicalTrials.gov NCT04007952. Registered on July 5, 2019.

## Administrative information

Note: the numbers in curly brackets in this protocol refer to SPIRIT checklist item numbers. The order of the items has been modified to group similar items (see http://www.equator-network.org/reporting-guidelines/spirit-2013-statement-defining-standard-protocol-items-for-clinical-trials/).

**Table:** 

**Title {1}**	Comparing Anterior Gastropexy to No Anterior Gastropexy for Paraesophageal Hernia Repair: A study protocol for a randomized control trial
**Trial registration {2a and 2b}.**	ClinicalTrials.gov NCT04007952. Registered on July 5, 2019. https://clinicaltrials.gov/ct2/show/NCT04007952?term=NCT04007952&cond=Paraesophageal+Hernia&draw=2&rank=1 Item 2b is not met. No items were collected from the World Health Organization Trial Registration Data Set.
**Protocol version {3}**	January 3, 2022. Version 1
**Funding {4}**	None.
**Author details {5a}**	K. E. Parnell^1^, S. J. Zolin^1^, C. Tu^2^, K. Baier^3^, L. R. Beffa^1^, D. Alaedeen^1^, D. M. Krpata^1^, A. S. Prabhu^1^, M. J. Rosen^1^, C. C. Petro^1^ ^1^Department of General Surgery, Digestive Disease and Surgery Institute, Cleveland Clinic Foundation, 9500 Euclid Avenue, A10-133, Cleveland, OH 44195, USA. ^2^Department of Quantitative Health Sciences, Cleveland Clinic Foundation, 9500 Euclid Avenue Cleveland, OH 44195, USA. ^3^Department of General Surgery at Fairview Hospital, Cleveland Clinic Foundation, 18101 Lorain Avenue, Cleveland, OH 44111, USA. Roles: K. E. Parnell^1^, S. J. Zolin^1^ – protocol authorship, data collection, management C. Tu^2^ – statistician, protocol authorship K. Baier^3^, L. R. Beffa^1^, D. Alaedeen^1^, D. M. Krpata^1^, A. S. Prabhu^1^, M. J. Rosen^1^ – Co-investigators, protocol authorship C. C. Petro^1^ – Primary investigator, protocol authorship
**Name and contact information for the trial sponsor {5b}**	Investigator initiated.
**Role of sponsor {5c}**	N/A. There is no sponsor for this trial.

## Introduction

### Background and rationale {6a}

Paraesophageal hernias occur when the stomach and/or other abdominal organs herniate through the diaphragmatic hiatus into the mediastinum. This abnormal anatomical configuration can lead to symptoms including acid reflux, dysphagia, and shortness of breath, and can also be a risk factor for gastric volvulus, a surgical emergency where blood flow to the stomach may be compromised. For these reasons, the Society of American Gastrointestinal and Endoscopic Surgeons (SAGES) has strongly recommended that all symptomatic paraesophageal hernias be repaired [1]. However, laparoscopic paraesophageal hernia repair (PEHR) presents a challenge for surgeons due to high rates of recurrence. The best available evidence suggests that more than half of patients undergoing laparoscopic PEHR will have radiographic hernia recurrence at 5 years after surgery [2]. Although the likelihood of patients needing revisional surgery for a recurrent hiatal hernia is low, ranging from 0.01 to 7% [2–10], patients with a recurrent hiatal hernia have increased symptoms of heartburn [8, 11–13], early satiety, gas bloat, difficulty and pain with swallowing [3, 4], and worse quality of life [14] compared to those without a recurrence. A prospective study by Le Page et al. evaluating 455 patients over 20 years after PEHR found that recurrences were also associated with increased rates of esophagitis [6]. For these reasons, several methods have been used during PEHR to reduce recurrence rates and improve symptoms. However, there remains substantial variability in techniques utilized, specifically for crural closure, and the use or avoidance of gastropexy, fundoplication, and/or mesh. A significant knowledge gap exists regarding which techniques are the most effective and whether or not they should be tailored for specific patients.

There has been suggestion in the scientific literature that use of anterior gastropexy—in which suture is used to affix the stomach to the anterior abdominal wall—may serve to reduce recurrence rates of PEHR [12, 15]. However, conflicting studies exist [16], and there is no consensus available from the surgical literature, which is limited to small, single-armed series, lacking control arms for comparison. The retrospective nature of this literature, with clear possibilities of selection bias and questionable generalizability, significantly limit a rigorous evaluation of the effect of anterior gastropexy on recurrence rates after laparoscopic PEHR. This leaves many surgeons reluctant to perform anterior gastropexy due to unclear long-term benefits coupled with the potential for short-term pain at the transfascial fixation site.

### Objectives {7}

The primary objective of this trial is to investigate the rates of radiographic PEH recurrence at 1 year in patients receiving anterior gastropexy compared to those receiving no anterior gastropexy during minimally invasive PEHR. The cumulative recurrence rates for laparoscopic PEHR with anterior gastropexy ranges from 0 to 25% [12, 15–20], compared to the recurrence rates without anterior gastropexy ranging from 7 to 66% [3, 16, 21–24]. This data is limited by variable lengths of follow-up and definitions of recurrences. We hypothesize that anterior gastropexy will result in a 15% reduction in PEH recurrence after minimally invasive repair compared to no gastropexy. Based on previously published data and our group consensus, we feel that a 15% reduction is a conservative but clinically important difference that supports the routine use of anterior gastropexy during PEHR.

Secondary objectives include the rates of symptomatic gastroesophageal reflux disease (GERD), dysphagia, odynophagia, gas bloat, regurgitation, chest pain, abdominal pain, nausea, vomiting, postprandial pain, cardiovascular, and pulmonary symptoms as well as patient satisfaction in the immediate postoperative and 1-year follow-up periods between the two groups.

### Trial design {8}

This is a multicenter, single-blinded, registry-based, randomized controlled, parallel group, superiority clinical trial.

## Methods: participants, interventions and outcomes

### Study setting {9}

This is a multicenter study involving two facilities within the Cleveland Clinic Foundation hospital system. The Cleveland Clinic Center for Abdominal Core Health, a center within the Digestive Disease and Surgery Institute (DDSI), at Cleveland Clinic Main Campus in Cleveland, Ohio, is the hosting department for the study. The second facility involved in the study is Cleveland Clinic Fairview Hospital in Cleveland, Ohio.

### Eligibility criteria {10}

The study population includes adult patients (≥ 18 years) undergoing elective, minimally invasive repair of a symptomatic, primary paraesophageal hernia measuring at least 5 cm in height on computed tomography (CT), upper gastrointestinal series (UGI), or endoscopy, where the diaphragmatic crura can be primarily reapproximated at the time of surgery without the use of mesh using either a laparoscopic or robotic approach. Only patients who are able to participate in follow-up and consent to participate will be included. Patients whose operations begin with a minimally invasive approach and are converted to open will be included provided that they meet all other criteria. The technique for crural closure and the use or avoidance of a fundoplication are both left to the discretion of the surgeon.

Exclusion criteria include patients who are not eligible for a minimally invasive repair or have undergone prior esophageal or gastric operations, including hiatal hernia repair, cruroplasty, esophageal lengthening procedures, esophagectomy, or any prior gastric resections. However, patients who have previously undergone gastrostomy tube placement alone will remain eligible for inclusion. Additional exclusion criteria include patients undergoing PEHR with a concurrent bariatric procedure (sleeve gastrectomy, Roux-en-Y gastric bypass, duodenal switch, single-anastomosis gastric bypass, and total gastrectomy), as well as patients undergoing PEHR with concomitant placement of a gastrostomy tube. If the surgeon feels mesh is necessary to augment hiatal closure at the time of operation or if a Collis gastroplasty is required for esophageal length, these patients will also be excluded. Reasons for patient exclusions from the trial will be documented and reported in the Consort diagram.

Surgeon eligibility requires fellowship training with expertise in minimally invasive PEHR. All participating surgeons are also required to undergo training to ensure understanding of the protocol and technical aspects of performing the anterior gastropexy as described in the protocol.

### Who will take informed consent? {26a}

Informed consent will be obtained by the study investigator or co-investigators during the preoperative visit.

### Additional consent provisions for collection and use of participant data and biological specimens {26b}

Not applicable. No additional consent provisions are needed.

#### Interventions

##### Explanation for the choice of comparators {6b}

The effect of anterior gastropexy on recurrence rates and patient-reported symptoms after PEHR is currently unknown, which limits surgeons’ ability to understand the clinical significance of this step. Therefore, anterior gastropexy (intervention arm) will be compared to no anterior gastropexy (control arm) in a prospective, randomized controlled study design.

##### Intervention description {11a}

Key steps of the operation are standardized. Skin preparation, hair removal, perioperative antibiotic administration, and venous thromboembolism prophylaxis are performed per Surgical Care Improvement Project protocol guidelines. Laparoscopic access to the abdomen is obtained by the operating surgeon through either Hasson or optical trocar entry depending on his or her standard practice. Additional laparoscopic ports are placed at the operating surgeon’s discretion to permit liver retraction, working ports, and assistant port(s) for additional retraction. The hernia sac is completely excised from the mediastinum and may be resected from the gastroesophageal junction per the discretion of the surgeon. If necessary for exposure, the short gastric vessels may be divided. The mediastinal esophagus is mobilized to allow for at least 3–4 cm of intraabdominal esophagus. After the surgeon is satisfied with the length and mobility of intraabdominal esophagus, the diaphragmatic crura are reapproximated with permanent suture. If Collis gastroplasty or mesh is deemed necessary, the patient will be excluded from the trial. However, fundoplication may be performed based on surgeon preference and indications, which will be documented.

After crural closure, the patient is randomized to the appropriate treatment arm. Randomization occurs in a 1:1 ratio using a computer-generated random allocation sequence to receive either no anterior gastropexy (control arm) or anterior gastropexy (intervention arm). A study coordinator and/or a research fellow is responsible for the randomization process.

If a patient is randomized to anterior gastropexy (intervention arm), this is performed as the next step of the procedure. Two 2-0 permanent sutures are placed in the midbody of the anterior stomach to provide fixation to the left upper quadrant abdominal wall below the costal margin. Two small incisions are made in the left upper quadrant to allow a suture passer to grasp the remaining ends of the sutures and externalize them from the abdomen at separate fascial punctures. The operation proceeds to the next step per individual surgeon practice. At the time of abdominal desufflation, the sutures are tied which fixates the stomach to the abdominal wall in this position.

After the diaphragmatic crura are reapproximated, if a patient is randomized to the control arm, the operation will proceed to the next step per individual surgeon practice without performing anterior gastropexy. Anterior gastropexy will be the main study intervention that differs between the two groups, which will allow for a direct comparison of patient outcomes attributable to this step.

##### Criteria for discontinuing or modifying allocated interventions {11b}

If at any point during the operation, the surgeon believes inclusion in the study is not in the patient’s best interest, the surgeon may choose to withdraw the patient from the study and complete the operation per his or her standard of care. However, if the patient is included in the study, there will be no deviation from the study protocol and surgeons must perform the key operative steps and technique for anterior gastropexy per the established study protocol. In the event of an immediate recurrence or other postoperative complication, if a gastropexy is added during a subsequent operation, the patient will be included in the intent-to-treat analysis.

As with any surgical procedure, patients may experience pain, bleeding, and discomfort. There is also a small risk of seroma, hematoma, inflammation, wound dehiscence, and infection at the surgical sites. After PEHR, there is a possibility of other symptoms including visceral injury, leak, nausea, vomiting, abdominal distention, dysphagia, odynophagia, GERD, and recurrent hiatal hernia. Patients in both study arms will receive appropriate clinical management for these symptoms at the discretion of the treating surgeon. If at any point during the postoperative period the surgeon believes gastropexy takedown or local suture removal is required to treat an ongoing symptom and/or is in the patient’s best interest, this may be performed at the surgeon’s discretion and will be documented.

##### Strategies to improve adherence to interventions {11c}

Since patients are not randomized until after crural closure, surgeon bias, and technical variations of the procedure will be avoided up to this point of the operation. Anterior gastropexy will be the main study intervention that differs between the two groups. All participating surgeons are fellowship-trained, specialize in minimally invasive surgery and have received appropriate training on the procedural steps required of the protocol. This expertise and training serve to minimize technical deviations of the protocol and yield reproducible results.

##### Relevant concomitant care permitted or prohibited during the trial {11d}

The surgical technique for paraesophageal hernia repair must follow study protocol; however, the perioperative care may vary and will be left to the discretion of the surgeon. Patients are permitted to seek pre- and postoperative medical care at Cleveland Clinic Foundation (CCF) and non-CCF institutions during the trial period.

##### Provisions for post-trial care {30}

Cleveland Clinic Center for Abdominal Core Health will continue to provide post-trial care and long-term follow-up for all patients participating in the trial. If any patient suffers a complication or harm from trial participation, CCF will provide appropriate medical and surgical care as per institutional standards. Adverse events will be reported to the IRB as appropriate. No preset compensation has been arranged for those who suffer harm from the trial.

### Outcomes {12}

#### Primary outcome

- The primary outcome of interest is radiographic paraesophageal hernia recurrence at 1 year postoperatively in patients receiving anterior gastropexy compared to those receiving no anterior gastropexy. An upper gastrointestinal series (UGI) or computed tomography (CT) scan will be obtained at the 1-year follow-up visit as per standard of care at our institution. Imaging obtained within the CCF system and at other institutions at the 1-year time point will be eligible for review provided that these are uploaded into Epic. Imaging will be reviewed by outcome assessors who are blinded to the treatment group and a consensus among assessors is required. A height of 2 cm of stomach, fundoplication or other abdominal organ (excluding the esophagus) above the diaphragm will constitute a radiographic recurrence, as previously described by Oelschlager et al. [25]. Herniation < 2 cm will not be considered a recurrence for the purposes of this study. Additionally, the need for reoperation secondary to fundoplication disruption, dysfunction, or slippage at any time during the study period will be considered a recurrence. We hypothesize that recurrence rates at 1 year after surgery for patients who receive anterior gastropexy will be decreased by 15% or more compared to those receiving no anterior gastropexy.

#### Secondary outcomes

- Patient-reported GERD, dysphagia, odynophagia, gas bloat symptoms, and patient satisfaction in the 30-day postoperative period and at 1-year follow-up will be measured by the GERD-Health-related Quality of Life symptom severity instrument (GERD-HRQL). GERD-HRQL is a validated 11-item instrument to quantify symptom severity of GERD, dysphagia, odynophagia, and gas bloat symptoms as well as patient satisfaction [26]..
- Additional symptoms, including regurgitation, chest pain, abdominal pain, nausea, vomiting, postprandial pain, cardiovascular, and pulmonary complaints in the 30-day postoperative period and at 1-year follow-up, will be measured by a numeric rating scale. Numeric rating scales have been used in prior outcomes research for PEHR [19, 22, 23] and measure patient symptoms experienced in the preceding 7 days.
- Other variables collected include postoperative wound and medical morbidity, length of stay, readmission, and patient-reported outcomes. These outcomes will be collected as standard of care in the Digestive Disease and Surgery Institute Quality Collaborative (DDSI-QC). Clavien-Dindo Classification and Comprehensive Complication Index, both validated tools [27, 28], will also be used to measure postoperative complications, morbidity and disease burden for each patient.

### Participant timeline {13}

Estimated patient accrual time is 4 years. Data collection will occur over 1 year from randomization of each patient, which includes the immediate 30-day postoperative and 1-year follow-up periods. Data analysis and manuscript production will occur within 6 months of the completion of data collection (Table 1).

**Table 1:** Schedule of enrolment, interventions, and assessments

	Study period			
	Enrolment	Allocation	Post-allocation	Close-out
**Timepoint**	**Preoperative period**	**Intraoperative**	**30-day follow-up**	**1-year follow-up**
**ENROLMENT:**				
**Eligibility screen**	**X**			
**Informed consent**	**X**			
**Allocation**		**X**		
**INTERVENTIONS:**				
**No gastropexy**		**X**		
**Gastropexy**		**X**		
**ASSESSMENTS:**				
**PEH recurrence**				**X**
**GERD**	**X**		**X**	**X**
**Dysphagia**	**X**		**X**	**X**
**Odynophagia**	**X**		**X**	**X**
**Gas bloat**	**X**		**X**	**X**
**Regurgitation**	**X**		**X**	**X**
**Chest pain**	**X**		**X**	**X**
**Abdominal pain**	**X**		**X**	**X**
**Nausea**	**X**		**X**	**X**
**Vomiting**	**X**		**X**	**X**
**Postprandial pain**	**X**		**X**	**X**
**Cardiovascular symptoms**	**X**		**X**	**X**
**Pulmonary symptoms**	**X**		**X**	**X**
**Patient satisfaction**			**X**	**X**
**Demographic data**	**X**			
**Operative details**		**X**		
**Wound morbidity**			**X**	
**Medical morbidity**			**X**	
**Length of stay**			**X**	
**Readmission**			**X**	**X**

*PEH* paraesophageal hernia, *GERD* gastroesophageal reflux disease

### Sample size {14}

Assuming a 20% rate of loss to follow-up, enrollment of 240 participants with 120 participants in each arm will provide at least 80% power for showing superiority of anterior gastropexy to no anterior gastropexy for the primary endpoint of decreased recurrence rates by 15% at the 1-year postoperative visit using a two-sided chi-square test and an alpha level of 5%. For the purpose of calculating the power of this study, the proportion of recurrences in the control group (no anterior gastropexy) is estimated to be 0.24. This is based on the reported recurrence rates for paraesophageal hernia repair without gastropexy, ranging from 7 to 66% [3, 16, 21–24], with the majority falling between 13 and 42% [3, 16, 23, 24] at various time points after surgery.

### Recruitment {15}

CCF hospital system is a quaternary care referral center and the DDSI performs approximately 80–100 PEHR annually. An accrual time of 4 years is estimated to be adequate to recruit 240 participants. Strategies for achieving adequate participant enrollment to reach target sample size includes offering all eligible patients seen in clinic the opportunity to participate. There remains a potential to expand this study to include other minimally invasive CCF surgeons with expertise in foregut surgery if needed for patient enrollment as well.

#### Assignment of interventions: allocation

##### Sequence generation {16a}

This is a two-armed trial with an allocation ratio of 1:1 between the control (no anterior gastropexy) and intervention (anterior gastropexy) arms. Patients are randomized using a computer-generated random allocation sequence. The random allocation sequence was built using a Research Electronic Data Capture (RedCAP®) database hosted at Cleveland Clinic Main Campus in Cleveland, Ohio. A randomization allocation table has been generated and uploaded into REDCap® by a CCF statistician. No planned restrictions will be included. Both institutions involved in this study will use the same randomization allocation sequence that is hosted at Cleveland Clinic Main Campus in Cleveland, Ohio.

##### Concealment mechanism {16b}

No concealment envelope or alternative mechanism is necessary since allocation will occur in the operating room when the patient is under general anesthesia. After crural reapproximation, surgeons at either institution will ask the circulating operating room nurse to call the study coordinator and/or a research fellow hosted at the Cleveland Clinic Main Campus. The study coordinator and/or a research fellow will generate the allocation sequence at this point and inform the operating room nurse and surgeon of the result.

##### Implementation {16c}

The participating surgeons (PI or Co-Is) and appropriate research personnel are responsible for enrollment during the preoperative clinic evaluation. A study coordinator and/or a research fellow is responsible for generating the allocation sequence intraoperatively per surgeon request after crural reapproximation.

#### Assignment of interventions: blinding

##### Who will be blinded {17a}

Patients are under general anesthesia when allocation assignments are distributed and thus blinded to their assigned intervention. Patients will remain blinded until their participation in the study is complete. The operating surgeons are unblinded to the allocation arm. However, patients are not randomized until crural closure is complete, thus limiting surgeon bias and technical variations of the procedure up to this point. Outcome assessors are blinded to the patients’ allocation arm when evaluating 30-day and 1-year follow-up data.

##### Procedure for unblinding if needed {17b}

Circumstances in which unblinding is permissible would be in the event of a postoperative surgical emergency that required revision of the recent paraesophageal hernia repair. The surgeon may disclose operative findings and events to the patient per their discretion.

#### Data collection and management

##### Plans for assessment and collection of outcomes {18a}

An UGI or CT scan will be used at the 1-year follow-up period to assess for radiographic hiatal hernia recurrence. Three surgeons will serve as the blinded assessors for this study and will complete training on the definition of PEH recurrence per the protocol. Agreement must be reached between at least two of them to be accepted as the consensus. GERD-HRQL, a validated 11-item instrument, will be used to assess GERD, dysphagia, odynophagia, and gas bloat symptoms as well as patient satisfaction at the 30-day and 1-year follow-up periods [26]. Additionally, numeric rating scales, which have been used in prior PEHR research [19, 22, 23], will be used to evaluate regurgitation, chest pain, abdominal pain, nausea, vomiting, postprandial pain, cardiovascular, and pulmonary symptoms at the 30-day and 1-year follow-up periods.

##### Plans to promote participant retention and complete follow-up {18b}

Patients will be scheduled for a postoperative clinic appointment within the 30-day and 1-year follow-up periods, both of which are our routine standard of care. Implementing 1-year radiographic follow-up will be the most challenging aspect of the trial. Several mechanisms have been established to promote patient retention and follow-up completion. The 30-day follow-up period will extend from 15 to 45 days postoperatively, and the 1-year follow-up period will extend from 6 to 18 months postoperatively. Appointment reminders will be sent via patient online portals and nursing phone calls. Virtual follow-up visits can be accommodated and patients can complete UGI or CT scans at their local institutions, provided the imaging can be uploaded into CCF’s digital platform for review. For patients who have missed follow-up appointments, the dedicated study coordinator, research fellow, and/or surgeon will call patients to reschedule or send the GERD-HRQL and numeric rating scales by mail to collect this information. This will increase convenience for patients and protocol adherence. Appropriate statistical analyses will be conducted to account for missing data.

##### Data management {19}

The DDSI-QC will serve as the platform for data collection, including baseline, follow-up, and outcomes data. It will also capture granular operative details. The DDSI-QC is an enterprise-wide quality improvement effort hosted at the Cleveland Clinic Foundation in the form of a database featuring prospectively collected, point-of-care, surgeon-entered data.

##### Confidentiality {27}

Subject anonymity and data confidentiality will be maintained before, during, and after the trial period. Every effort will be made to maintain the confidentiality of documents that identify the subject by name (e.g., signed informed consent documents, clinic charts), except to the extent necessary to allow monitoring by the Office of Research Compliance at the Cleveland Clinic or by other regulatory authorities.

The information collected will be stored in the DDSI-QC, a secure institutional database that is used to track clinical outcomes in patients who undergo operations by surgeons in CCF’s DDSI. Written consent forms and data collection forms will be stored in binders, which will stay in a locked office in the Crile Building at CCF. Randomization will occur with the use of a customized REDCap®, a secure network/firewall-protected electronic database to which only the investigator and the designated members of the study team will have access using an individually assigned login and password. Only approved study members listed on the Institutional Review Board (IRB) protocol will have access to the separately stored master list—which will be stored in a Cleveland Clinic password-protected computer and saved on an S drive. Only the Principal Investigator, lead research coordinators, and biostatisticians will be granted access to retrieve patient data for data quality assessment and data analysis. All electronic records pertaining to the clinical study will be password-protected and only approved study members listed on the IRB protocol will have password access.

##### Plans for collection, laboratory evaluation, and storage of biological specimens for genetic or molecular analysis in this trial/future use {33}

Not applicable. No biologic specimens will be collected during the study.

#### Statistical methods

##### Statistical methods for primary and secondary outcomes {20a}

All analyses will be performed using the intent-to-treat population and will be done under the normality assumption, if appropriate. Patient characteristics will be summarized overall and by randomized group, and differences will be described as standardized effects. All tests will be considered significant at 5% level. Outliers for each endpoint will be assessed with a graphical method, such as the box plot. All primary and secondary endpoints will be initially evaluated using all observations and then evaluated with a sensitivity analysis without including points identified as outliers. All statistical analyses will be performed with SAS software (version 9.4; Cary, NA) or R software (version 4.0.0, 2020-04-24; Vienna, Austria).

##### Primary outcome

The primary endpoint of this study is radiographic recurrence rates at 1 year following either anterior gastropexy (intervention) or no anterior gastropexy (control) during paraesophageal hernia repair. Differences between the two groups will be tested and described using a binomial regression model with identity link and robust variance estimation. Pre-specified covariates will include fundoplication vs. no fundoplication, Type II/III vs. Type IV PEH, body mass index (BMI), sex, and surgeon, with the surgeons modeled as cluster effect. Results will be presented as relative risk and absolute risk differences with a 95% confidence interval. In cases where the model fails to run due to convergence issues, a Poisson GEE model with robust variance estimates, a log Poisson regression model, or a linear regression GEE model with exchangeable correlation and robust standard errors will be used to estimate the risk difference.

##### Secondary outcomes

Secondary outcomes include patient-reported symptoms of GERD, dysphagia, odynophagia, gas bloat, regurgitation, chest pain, abdominal pain, nausea, vomiting, postprandial pain, cardiovascular, and pulmonary complaints along with patient satisfaction from GERD-HRQL and the numeric rating scale measured at baseline, 30 days postoperatively, and 1 year postoperatively. Generalized linear mixed effect models will be used to compare categorical outcomes, and linear mixed effect models will be used to compare numeric continuous score outcomes. In these models, groups will be analyzed using fixed effect parameters and surgeons will be analyzed using random effect parameters.

### Interim analyses {21b}

No interim analyses will be performed.

### Methods for additional analyses (e.g., subgroup analyses) {20b}

A priori defined exploratory analyses will be performed to evaluate the effects of fundoplication on symptoms of GERD, dysphagia, regurgitation, and chest and abdominal pain. For patients with PEH recurrence, the differences in symptoms of those with a fundoplication will be compared to those without a fundoplication as another exploratory endpoint. Our a priori hypothesis is that a recurrent hiatal hernia with a fundoplication in the mediastinum is more symptomatic for patients than recurrences without a fundoplication in the mediastinum. Differences between groups will be tested and described as standardized effects using chi-square test or Fisher’s exact test for binary variables and Student’s *t* test or Wilcoxon rank sum test for continuous variables with a 95% confidence interval and a *P* value of <0.05 denoting statistical significance.

### Methods in analysis to handle protocol non-adherence and any statistical methods to handle missing data {20c}

Missing data over time is anticipated to reflect a pattern of “missing at random.” Data collection at the time of the initial surgical consultation (enrolment period) and during the operation (randomization period) will be negligible, as we will ensure capture of all necessary measurements during these time points. If necessary, multiple imputation will be used to impute missing baseline data. Provided that the “missing at random” assumption holds true, multiple imputation of follow-up measurements will not be performed. Since this study involves surgical intervention at the time of randomization, non-adherence to the intervention is not expected to occur.

For missing data during the follow-up period, mixed effect models will be used, which by virtue of their maximum likelihood approach will provide reliable estimates when missing data are “missing at random.” This assumption will be graphically evaluated, and if necessary, sensitivity analyses will be performed to assess the robustness of our findings. This approach will be used for all secondary endpoints.

As for the primary endpoint, obtaining 1-year radiographic assessments will be the most challenging aspect of this study. Several mechanisms are in place to account for missing radiologic data. First, a 20% loss to follow-up rate is expected and was incorporated into the sample size calculation during study development. Second, “intent-to-treat” analysis will be performed, and the “last value carried forward” method will be applied to missing observations. In this method, patients who are lost to follow-up will be considered as “no recurrence,” and the total patient population of 240 will remain as the denominator. Lastly, tipping point analysis will be performed as a sensitivity analysis to examine how missing 1-year radiographic follow-up data affects the validity of our findings.

If non-random missing data patterns are observed, alternate sensitivity analyses, such as pattern mixture models, will be considered.

### Plans to give access to the full protocol, participant-level data, and statistical code {31c}

There are no plans for granting public access to the full protocol, participant-level dataset, and statistical code.

The SPIRIT reporting guidelines were used during the production of this manuscript and can be referenced here:

Chan A-W, Tetzlaff JM, Gøtzsche PC, Altman DG, Mann H, Berlin J, Dickersin K, Hróbjartsson A, Schulz KF, Parulekar WR, Krleža-Jerić K, Laupacis A, Moher D. SPIRIT 2013 Explanation and Elaboration: Guidance for protocols of clinical trials. BMJ. 2013;346:e7586.

#### Oversight and monitoring

##### Composition of the coordinating center and trial steering committee {5d}

Study coordination and trial steering will be carried out by the Cleveland Clinic Center for Abdominal Core Health research team within the DDSI department at Cleveland Clinic Foundation, Cleveland, Ohio. The research team consists of several faculty surgeons who serve as either the PI or Co-I’s, one clinical research nurse and several research fellows, residents, and medical students. The entire research team meets weekly to evaluate study progression, adverse events, and any updates to study procedures and requirements. The research team performs the functions of a steering committee, endpoint adjudication committee, data management team, and oversight team. Day-to-day trial organization and data management are the responsibility of the dedicated research residents, fellows, and clinical nurse on the research team. The PI and Co-I’s will oversee the progression of the study, ensure adherence to protocol, and provide guidance with publication.

##### Composition of the data monitoring committee, its role and reporting structure {21a}

A data monitoring committee is not necessary for this study since both allocation arms, gastropexy, and no gastropexy, have already been established as standard of care and safe in the surgical community for paraesophageal hernia repair. The efficacy of anterior gastropexy is uncertain, not the safety, which is why it is regarded as an optional step of the operation based on surgeon discretion.

##### Adverse event reporting and harms {22}

Adverse events will be promptly reported to the IRB. Either the IRB or clinical research team may recommend prematurely ending the trial following clear evidence of patient benefit or harm.

##### Frequency and plans for auditing trial conduct {23}

The research team and study investigators will evaluate trial conduct on a weekly basis. In addition, the DDSI Research Infrastructure and Regulatory Team oversees all research studies in the DDSI department and periodically audits each trial per random selection throughout the study period. This trial was last audited September 2021.

##### Plans for communicating important protocol amendments to relevant parties (e.g., trial participants, ethical committees) {25}

Important protocol modifications, including changes to eligibility criteria, outcomes, and analyses, will be promptly communicated to all relevant parties in formal writing per the discretion of the research team.

##### Dissemination plans {31a}

The trial results will be communicated to healthcare professionals via publication. After the study period has concluded, patients may become unblinded to their treatment arm and made aware if they received anterior gastropexy or no anterior gastropexy. The target audience is healthcare professionals; therefore, no formal efforts will be made to inform patients of the study results.

## Discussion

This randomized controlled trial will assess the effects of anterior gastropexy on medium-term recurrence rates and patient-reported outcomes after paraesophageal hernia repair. The use of anterior gastropexy has a theoretical benefit of decreasing paraesophageal hernia recurrence rates. However, this has not been proven beyond a suggestion of effectiveness in retrospective research. Although the likelihood of patients needing revisional surgery for a recurrent hiatal hernia is low, radiographic recurrence may portend risk for further symptoms and/or reoperation due to the anatomical abnormality. Radiographic recurrence is also the most objective measure of success of the operation. If the use of anterior gastropexy proves to reduce recurrence rates, this finding would help with operative decision-making and surgical management of these hernias. If anterior gastropexy proves to be ineffective, this step may be abandoned due to futility and patient discomfort at the transfascial fixation points.

Anterior gastropexy was chosen as the intervention arm of this trial since there is suggestion in the literature that it may serve to reduce recurrence rates of PEHR. Ponsky et al. described a series of 28 PEHR in which anterior gastropexy was used routinely and no recurrences were noted at 1 year postoperatively [15]. Subsequently, Poncet described a series of 89 patients undergoing PEHR in which 86.5% underwent anterior gastropexy and recurrence rates at a mean duration of follow-up exceeding 4 years were 10.8% in the anterior gastropexy group versus 50% in the group without gastropexy [12]. In contrast, Diaz described selective use of anterior gastropexy in a series of 116 patients undergoing PEHR, primarily for patients with preexisting gastric volvulus (*n* = 48), and at 30 months mean follow-up reported a higher recurrence rate of 25% in the anterior gastropexy group compared to 13% in those without anterior gastropexy [16]. The retrospective nature of this literature, with clear possibilities of selection bias and questionable generalizability, significantly limit a rigorous evaluation of the effect of anterior gastropexy on recurrence rates after laparoscopic PEHR.

Anterior gastropexy, along with several other technical steps, serve as branch points during operative decision-making which may affect outcomes of PEHR. These include, but are not limited to the following: minimally invasive versus open approaches, various crural closure techniques, addition or avoidance of fundoplication, mesh use versus no mesh use, and the use or avoidance of anterior gastropexy. We chose to exclude patients with mesh at the hiatus because there is evidence that it, at most, delays early recurrence of paraesophageal hernias [2, 25]. Our primary endpoint of PEH recurrence at 1 year ± 6 months is a short to medium-term follow-up period, and mesh placement would likely be a confounder if included. Patients not eligible for a minimally invasive repair were similarly excluded since there is evidence, albeit retrospective in nature, that open PEH repair has lower recurrence rates compared to laparoscopic approaches [24, 29]. On the other hand, there is no evidence to suggest that a fundoplication affects recurrence rates after paraesophageal hernia repair [30, 31]. Therefore, fundoplications were allowed in this protocol per surgeon discretion and are not expected to confound the primary endpoint. We will analyze this assumption at the conclusion of the study with a multivariate logistic regression model in patients with and without a fundoplication. We also hypothesize that a recurrent hiatal hernia with a fundoplication in the mediastinum is more symptomatic than recurrences without a fundoplication in the mediastinum. A priori exploratory outcome analyses will be performed to examine this hypothesis. Since there is no evidence to show that a fundoplication decreases recurrence rates, and it has the potential for increased symptoms of dysphagia, regurgitation, and chest pain after recurrence, it is currently not our standard practice to routinely perform a fundoplication during paraesophageal hernia repair. This study will elucidate these hypotheses and help power a larger study for a more formal evaluation. Patients who have undergone a prior paraesophageal hernia repair, cruroplasty, or anti-reflux procedure or have had esophageal or gastric resections in the past were excluded due to the inability to control for the variable anatomy at the hiatus which could confound resultant effects on recurrence rates and patient-reported symptoms. For similar reasons, if a Collis gastroplasty is needed for a shortened esophagus, these patients are not randomized. Our group rarely performs this procedure and if needed, the anatomic variation in this scenario would not be representative of the standard patient in this study population.

### Trial status

Protocol version 1, January 3, 2022. Currently Recruiting. Recruitment began on June 26, 2019, and is expected to be completed July 1, 2023.

## Data Availability

Only approved study members listed on the IRB protocol will have access to the separately stored master list—which will be stored in a Cleveland Clinic password-protected computer and saved on an S drive. Only the Principal Investigator, lead research coordinators, and biostatisticians will be granted access to retrieve patient data for data quality assessment and data analysis. All electronic records pertaining to the clinical study will be password-protected and only approved study members listed on the IRB protocol will have password access.

## Abbreviations

PEHR: Paraesophageal hernia repair
GERD: Gastroesophageal reflux disease
UGI: Upper gastrointestinal series
CT: Computed tomography
GERD-HRQL: GERD-Health-Related Quality of Life Symptom Severity Instrument
DDSI-QC: Digestive Disease and Surgery Institute Quality Collaborative
RedCAP®: Research Electronic Data Capture
CCF: Cleveland Clinic Foundation
IRB: Institutional Review Board
ASA: American Society of Anesthesiologists class
BMI: Body mass index
DSMB: Data Safety Monitoring Board
SAGES: Society of American Gastrointestinal and Endoscopic Surgeons
